# Distribution of GNAQ and GNA11 Mutation Signatures in Uveal Melanoma Points to a Light Dependent Mutation Mechanism

**DOI:** 10.1371/journal.pone.0138002

**Published:** 2015-09-14

**Authors:** Mark J. de Lange, Lubna Razzaq, Mieke Versluis, Sven Verlinde, Mehmet Dogrusöz, Stefan Böhringer, Marina Marinkovic, Gregorius P. M. Luyten, Rob J. W. de Keizer, Frank R. de Gruijl, Martine J. Jager, Pieter A. van der Velden

**Affiliations:** 1 Department of Ophthalmology, Leiden University Medical Center, Leiden, the Netherlands; 2 Department of Medical Statistics, Leiden University Medical Center, Leiden, the Netherlands; 3 Department of Dermatology, Leiden University Medical Center, Leiden, the Netherlands; University of Alabama at Birmingham, UNITED STATES

## Abstract

Uveal melanomas (UM) originate from melanocytes in the interior wall of the eye, namely from the iris, ciliary body and the choroid with marked differences in light exposure (from dark anterior to illuminated posterior). In contrast to UV radiation, focused or converging visible light readily reaches the retina and can damage DNA which possibly contributes to UM development. In this report choroidal, ciliochoroidal and iridociliary melanomas were analyzed for GNAQ and GNA11 mutations which were subsequently correlated to the location of tumor origin. Hotspot mutations in GNAQ and GNA11 can be divided in A>T and in A>C mutation signatures. The GNAQ A626C mutation (Q209P) was almost exclusively observed in choroidal melanomas from the illuminated posterior side. On the other hand, ciliochoroidal UM from the dark anterior side with mostly A>T mutations were clearly associated with light-colored eyes. Combined these data suggest a light and a pigment dependent etiology in UM development.

## Introduction

Uveal melanoma (UM) is the most common primary intraocular malignant tumor and can be divided into posterior tumors, located in the choroid and/or the posterior part of the ciliary body, or anterior tumors [1,2]. Anterior segment melanomas are divided into iris melanomas with and without anterior chamber angle extension, and iridociliary melanomas which involve the iris and the anterior ciliary body. Iris and iridociliary melanomas account for 3–10% of all uveal melanomas and are the most common primary malignancy of the iris [3,4].

Melanocytes that give rise to melanoma in the skin and the uvea belong to distinct subtypes that involve different developmental and regulatory pathways [5]. Hence it is not surprising that the origin of UM is different from cutaneous melanoma (CM). Whereas mutations in the BRAF and NRAS genes have been shown to be present in most of the CM, GNAQ and GNA11 mutations are involved in the development of UM [6–12]. The mechanisms that generate the mutations in CM and UM are largely unknown. The BRAF hotspot mutation in CM (1799T>A V600E) does not bear the typical “UV signature” (C>T in dipyrimidine sites) and therefore does not reflect sun exposure as a risk factor [13]. Because homologous base substitutions (i.e. A>T and T>A) are observed in BRAF, GNA11 and GNAQ, a common mechanism may lead to these mutations. Sunlight exposure may be a shared factor in melanoma development though the role of sunlight exposure is even more controversial in UM than in CM. Some epidemiological studies support a role for sunlight exposure in the pathogenesis of UM while others fail to show a correlation [14–16]. Because solar light exposure differs between the iris, ciliary body and choroid, UM provides a model to study (solar) light induced mutagenesis in melanocytes distributed over the interior wall of the eye. Alternatively, light pigment synthesis provides a melanocyte-specific endogenous source of DNA damage as fair skin and light eye color are both risk factors for CM and UM [17]. In order to investigate pigment synthesis and light exposure as underlying mechanisms in UM development we have compared the site of origin and the patient’s eye color with the GNAQ/GNA11 mutation profile in a series of enucleated UM. Based on the unique anatomy of the eye we are able to distinguish light-dependent and pigment-dependent etiologies.

## Materials and Methods

### Patient Selection

Written informed consent was obtained and this study was approved by the Medical Ethics Committee (CME) of the Leiden University Medical Center. The tumors in this study were restricted to choroidal melanoma, choroidal melanoma with ciliary body extension (hereafter termed ciliochoroidal melanoma) and melanoma that involved the iris with or without ciliary body involvement (hereafter termed iridociliary melanoma). Choroidal melanoma and ciliochoroidal melanoma tissue was obtained from enucleation specimens. Clinical and pathological charts were used to obtain information on tumor location, tumor characteristics and eye colour. Central was defined as juxtapapillary and within the vessel arcade, midperipheral as between the vessel arcade and the equator, and peripheral as anterior to the equator. Large tumors could span multiple areas. Sections of fresh-frozen tissue were used for analysis. For iris and iridochoroidal melanoma, paraffin-embedded tissue sections were retrieved from the pathology department.

### Tissue dissection and DNA extraction

Choroidal and ciliochoroidal melanoma DNA was isolated from sections of fresh frozen tissue. Tissue samples of iris samples were obtained by macro dissection of 10 μm sections from formaldehyde-fixed paraffin-embedded samples.

DNA was extracted from the tumor tissue sections using either the QIAamp DNA minikit from Qiagen (Venlo, Netherlands) in the case of freshly frozen tissue or the RecoverAll ™ Total Nucleic Acid Isolation Kit (Life Technology, Bleiswijk, Netherlands) for paraffin-embedded tissue.

### BRAF, GNAQ and GNA 11 sequencing

GNAQ, GNA11 and BRAF amplicons were attained from UM by PCR using Sybr green premixture (Bio-Rad, Veenendaal, Netherlands). The following protocol was used for amplification of exon 5 of the GNA11 and the GNAQ genes:

94°C, 3min; (96°C, 15sec; 63°C, 15sec; 72°C, 1min) 7X; (96°C, 15sec; 61°C, 15sec; 71°C, 1min) 8X; (96°C, 15sec; 60°C, 15sec; 72°C, 1min), 36X; 72°C, 1min, end. For amplification of exon 15 of BRAF the following protocol was used:

94°C, 3min; (96°C, 15sec; 60°C, 15sec; 72°C, 30sec) 40X; 72°C, 1min, end.

The primers used in PCR consisted of:

CGCTGTGTCCTTTCAGGATGGTG, GNA11ex5F

GCCCACCTAGTTGTCCGACT, GNA11ex5R

CCCTAAGTTTGTAAGTAGTGCTATATTTATGTTG, GNAQex5F

ATGATAATCCATTGCCTGTCTAAAGAACAC, GNAQex5R

AACTCTTCATAATGCTTGCTCTGATAGG, BRAFex15F

GCCTCAATTCTTACCATCCACAAAATG, BRAFex15R

After amplification, DNA clean-up was performed using the Nucleospin Extract II columns of Macherey-Nagel (Düren, Germany) following the manufacturer’s instructions. For sequencing analysis, samples were prepared by adding 10 pmol of the forward or the reverse primer to the purified DNA amplicon. Sequencing for mutations was performed at the Leiden Genomic Technology Center (LGTC) of the LUMC.

### Statistical analysis

Chi square test was used for statistical analysis of mutation distribution. Likelihood ratios were used for cases in which the Chi square assumption was violated.

## Results

**Fig 1:**
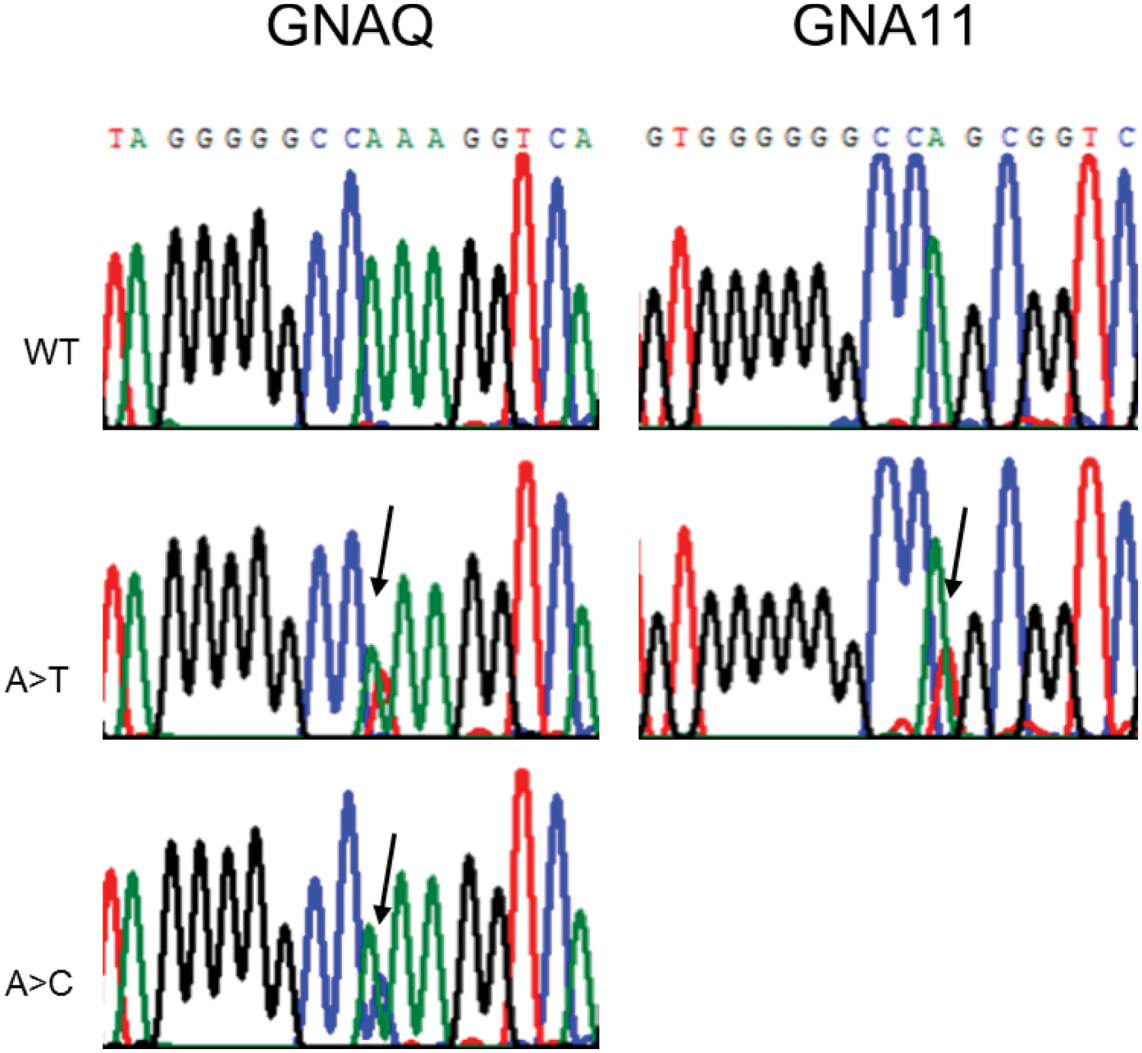
Sequence analysis of the GNAQ Q209P (A>C), GNAQ Q209L (A>T) and GNA11Q209L (A>T) hotspot mutations in UM.

In order to correlate mutation type with eye color and location of origin we characterized 133 UM (Table 1). We were able to obtain mutational status of 123 tumors out of which 94% contained either a GNAQ (48%) or GNA11 (46%) mutation covering all locations in the eye (Fig 1, Table 2). Using direct sequencing, neither BRAF nor NRAS mutations were identified in the UM samples (data not shown).

**Table 1:** Patient characteristics.

Clinical parameters	Count	Missing	Percentage
UM patients	133	0	100
Gender		0	100
Male	70		52.6
Female	63		47.4
Location	111	22	83.5
Choroidal	64		48.1
Ciliochoroidal	35		26.3
Iridociliary	12		9.0
Zone	100	33	75.2
Peripheral	52		39.1
Mid-peripheral	29		21.8
Juxtapapillary	14		10.5
Central	5		3.8
Eye color	68	65	51.1
Blue	30		22.6
Grey	13		9.8
Brown	11		8.3
Green	14		10.5
Lipofuscin	44	89	33.1
Yes	19		14.3
No	25		18.8

Frequencies of relevant parameters of 133 UM

**Table 2:** Distribution of mutational status.

Clinical parameters	Mutational status						
	WT	G11_QL	GQ_QL	GQ_QP	GQ_QH	Total	p-value
Location							
Choroidal	3	26	6	23	0	58	0.001
Ciliochoroidal	1	22	6	6	0	35	
Iridociliary	3	3	4	0	1	11	
Total	7	51	16	29	1	104	
Eye color							
Blue/grey	2	19	12	6	1	40	0.002
Brown/green	5	13	0	6	0	24	
Total	7	32	12	12	1	64	

UM GNAQ and GNA11 mutation status varies with tumor location and eye color. The GNAQ Q209P allele is almost uniquely found in UM originating from the central/choroidal area that is exposed to focused visible light. The GNAQ Q209L mutation, on the other hand, is correlated with light eyes (blue/grey).

### Mutation signature is correlated with anatomic location

In order to investigate GNAQ and GNA11 mutation, according to anatomic location, UM were grouped in choroidal melanoma, ciliochoroidal melanoma and iridociliary melanoma. Ciliochoroidal tumors originate either from the ciliary body or from the choroid with its extension towards the ciliary body. Similarly iridocialiary melanoma originate from the iris with a possible ciliary body involvement. GNAQ mutations were found in choroid, ciliochoroidal and iridociliary melanoma in respectively 50%, 34% and 45% of the cases. GNA11 mutations were found in choroid, ciliochoroidal and iridociliary melanoma in respectively 45%, 63% and 27% of the cases per location. Differences were however revealed when the mutations were analyzed separately. When we subdivided UM based on type of mutation, GNA11 Q209L and GNAQ Q209L were equally distributed over all locations in the eye. However, out of 29 UM (28%) that presented the GNAQ Q209P, 23 (79%) were located in the choroid (Table 2). Subsequently, we subdivided UM based on type of base substitution as A>T and A>C underlie all GNAQ and GNA11 mutations. In total 67 UM presented an A>T (GNAQ/11 Q209L) mutation and 30 UM presented an A>C mutation (GNAQ Q209P/Q209H). Localization in the choroid correlated with the A>C mutation (p = 0.028). The choroid melanomas that present the A>C mutation originate from the light-exposed area of the eye.

Light induced retinal damage is correlated with lipofuscin and therefore we also correlated the mutations with this marker. Half (53%) of the UM with lipofuscin tested positive for the A>C mutation while approximately a quarter of the lipofuscin negative UM presented the A>C mutation (p = 0.078) (Table 3).

**Table 3:** Distribution of mutation signatures.

Clinical parameters	Mutational status			
	A → T	A → C	Total	p-value
Location				
Choroidal	32	23	55	0.028
Ciliochoroidal	28	6	34	
Iridociliary	7	1	8	
Total	67	30	97	
Eye color				
Blue/grey	31	7	38	0.272
Brown/green	13	6	19	
Total	44	13	57	
Lipofuscin				
Yes	9	10	19	0.078
No	17	6	23	
Total	23	19	42	

When subdividing GNAQ/11 mutation signatures, A>C mutations were predominantly found in in the choroid as opposed to A>T mutations which were not location dependent. The presence of lipofuscin in A>C signature tumors supports the hypothesis that A>C mutations are likely to be caused by solar light damage, however this did not reach statistical significance.

### Eye color predisposes for UM location

We obtained eye color information from 68 patients which we could correlate to mutational status and location. Thirty patients presented blue eyes, thirteen had grey eyes, eleven had brown eyes and fourteen had green eyes. For statistical analysis, we combined blue and grey eyes to represent light eyes and brown and green eyes to represent dark eyes. Light eye color was linked to UM from all locations but was mostly correlated with ciliochoroidal melanoma, which were rarely found in dark eyes (p = 0.020, Table 4). Remarkably, UM bearing the GNAQ Q209L mutation were most commonly found in light eyes (p = 0.002) but this did not hold for the GNA11 Q209L mutation which is characterized by the same base substitution (A>T) (Table 2).

**Table 4:** Relationship of location and eye color.

Clinical parameters	Location				
	Choroidal	Ciliochoroidal	Iris	Total	p-value
Eye color					
Blue/grey	14	19	6	39	0.025
Brown/green	18	5	3	26	
Total	32	24	9	65	

Light eye color in UM patients reflects the population distribution though choroidal UM may present the lower estimate of light eye color while ciliochoroidal and iridociliary UM present the upper limit of the population frequency.

## Discussion

UM is a rare tumor for which only recently the first step in the molecular etiology was revealed with the identification of GNAQ and GNA11 mutations [11,12]. Development of uveal melanocytes is regulated by the EDNRB pathway that is correlated with Gαq-protein (GNAQ/GNA11) activation [5]. Apparently, the regulation of uveal melanocytes determines oncogene dependence of UM on mutant GNAQ/GNA11, in contrast to mutant BRAF/NRAS in CM. The molecular mechanisms that lead to GNAQ and GNA11 mutations are, however, unknown. Epidemiologic and clinical data suggests a role for solar light exposure in UM development but these data have been inconclusive [14–16].

We report that the GNAQ Q209P mutation is almost exclusively detected in choroidal melanoma that originate from the posterior region. This is the region in the eye that is most exposed to focused light and we therefore hypothesize that damaging visible light is involved in choroidal melanoma development. Especially blue light is suspected to cause damage and may contribute to the occupational UM risk that is reported for welders [18].

Lipofuscin deposition is a marker for retinal light damage that is found in choroidal melanoma and in uveal nevi that are at risk of progression to melanoma [19]. Lipofuscin deposition in choroidal melanoma thereby marks light-induced damage and represents a possible cause of DNA mutations by way of the activated A2E component [20]. A2E induced 8-oxo-deoxyguanosine that is incorporated in DNA replication, may lead to the GNAQ Q209P (A>C) mutation in a successive round of replication [21]. Epidemiologic research indicates that other mechanisms should exist that give rise to A>C mutations. In esophageal adenocarcinoma the A>C mutation was the most common mutation signature which has a homologous context to GNAQ [22]. The mutation is correlated with acid reflux disease but no specific carcinogenic compound has been identified. Possibly, oxidative stress gives rise to an A>C transversion by way of hydroxyl adenine in DNA [23]. As lipofuscin and focused visible light (blue light in particular) are unique to the macular region it may explain the preferential occurrence of the Q209P mutation in choroidal melanoma. The role of lipofuscin depositions in UM development should ideally be studied clinically in high-risk uveal nevi and correlated with the GNAQ/GNA11 mutations in the succeeding UM.

UM are correlated with light eyes in all anatomic locations of the eye but ciliochoroidal melanoma development is most correlated with light eye color (Table 2). This suggests that the risk of light eyes is independent of light exposure and this hypothesis is supported by a recent report in which CM development was shown to be correlated with intrinsic properties of light pigment (pheomelanin) in the skin [17]. An underlying mechanism could be that the synthesis of pheomelanin is associated with genotoxic stress. S-adenosyl methionine is part of the methionine cycle and feeds pheomelanin synthesis with cysteine. S-adenosyl methionine can also act as methyl donor in DNA-alkylating reactions and can cause A>T transversions due to methyl-adenosine adduct formation [24]. The observation that ciliochoroidal melanoma is almost exclusively correlated with A>T mutations in GNAQ Q209L and GNA11 Q209L (16/21) in patients with light eyes (11/14) supports this hypothesis (Tables 2 and 3). Possibly the same mechanism also generates the BRAF V600E (T-A) mutation that was detected in UM as minority allele [25,26]. Whether these proposed mutation mechanisms cause UM development should ultimately be studied in (humanized) animal models [17]. Besides a role in mutagenesis, melanogenesis in the melanocyte affects many cellular mechanisms [27]. In CM, melanogenesis regulates cellular metabolism via HIF-1α and pigmentation was found to be correlated with clinical outcome [28,29]. Whether melanogenesis plays the same role in UM is questionable as melanocyte development and regulation differs between skin and uvea [5]. Moreover, pigment in the skin is solely produced by melanocytes while in the eye the retinal pigment epithelium (RPE) also contains pigment [30].

Combined, GNAQ and GNA11 mutation distribution analysis suggests both location-specific and generic mutation mechanisms in UM. We propose that light-induced damage may underlie the almost exclusive presence of the GNAQ Q209P mutation in choroidal melanoma. Pheomelanin synthesis on the other hand may represent a more generic mechanism in melanogenesis that is involved in UM development all over the uvea and that can be crucial for UM development in anatomic locations that are not exposed to light.
